# High Cancer Susceptibility Candidate 8 Expression Is Associated With Poor Prognosis of Pancreatic Adenocarcinoma: Validated Analysis Based on Four Cancer Databases

**DOI:** 10.3389/fcell.2020.00392

**Published:** 2020-06-04

**Authors:** Yingyi Wang, Yuemei Yang, Yanfeng Wang, Xiaoou Li, Yu Xiao, Wenze Wang

**Affiliations:** ^1^Department of Oncology, Peking Union Medical College Hospital, Chinese Academy of Medical Sciences and Peking Union Medical College, Beijing, China; ^2^Molecular Pathology Research Center, Peking Union Medical College Hospital, Chinese Academy of Medical Sciences and Peking Union Medical College, Beijing, China; ^3^Department of Pathology, Heilongjiang Province Land Reclamation Headquarter General Hospital, Harbin, China; ^4^Department of Pathology, Daxing Hospital Affiliated to Capital Medical University, Beijing, China; ^5^Department of Pathology, Peking Union Medical College Hospital, Chinese Academy of Medical Sciences and Peking Union Medical College, Beijing, China

**Keywords:** long non-coding RNA, cancer susceptibility candidate 8, pancreatic adenocarcinoma, competing endogenous RNA, miRNA

## Abstract

**Objective:**

The aim of this study was to explore the association between the expression of a long non-coding RNA (lncRNA), cancer susceptibility candidate 8 (CASC8), and pancreatic adenocarcinoma (PAAD).

**Materials and Methods:**

starBase database was used to perform differential expression, survival, and competing endogenous RNA (ceRNA) network and H19/miR-671 correlation analyses for CASC8 in 178 PAAD samples. Using the cBioPortal database website, we analyzed the alteration in CASC8 expression and its correlation with the overall survival in PAAD. Gene Ontology (GO) and Kyoto Encyclopedia of Genes and Genomes (KEGG) enrichment analyses were also performed using the circlncRNAnet database. Analysis of CASC8 polymorphisms was performed using the UCSC Xena database. Finally, the expression of CASC8 in Chinese PAAD tissues was validated by qPCR.

**Results:**

The expression of CASC8 was observed to be high in 178 PAAD samples [fold change = 8.71, *P* = 0.0014, false discovery rate (FDR) = 0.04] and was related with poor prognosis, but not in pancreatic neuroendocrine tumor (pNET). CASC8 amplification was noted in 6% of the PAAD patients; however, the gene amplification did not affect the expression of CASC8 but was involved with the overall survival time of PAAD patients. Network analysis indicated that H19 is the ceRNA pair of CASC8 and that CASC8 competitively binds to miR-671 and might participate in the process of epithelial-to-mesenchymal transition (EMT). The correlation analysis showed that CASC8 was significantly negatively correlated with SMAD7. The analysis of CASC8 polymorphism showed that high copy number segment (CNS) of CASC8 is associated with low survival. Validation using PAAD tissues from Chinese patients was consistent with the *in silico* findings.

**Conclusion:**

CASC8 is specifically expressed at a high level in PAAD and associated with poor prognosis, which might be through its interaction with H19, miR-671, and SMAD7. These results indicate that CASC8 could serve as a novel marker for predicting the prognosis and as a potential target for the therapy of PAAD.

## Introduction

Pancreatic adenocarcinoma (PAAD) is one of the tumors with the highest morbidity and mortality (Siegel et al., 2020). Presently, surgical resection is the main treatment for early stage PAAD; however, effective therapies for late stage cases are lacking. Overall, PAAD has a very poor prognosis, with no more than 25% of PAAD patients surviving 1 year and only about 5% of the patients live for 5 years after diagnosis (Canada et al., 2016; Mcguire, 2016). For early-stage cancers, the 5-year survival rate rises to about 20%. However, no symptoms or diagnostic markers are specific to PAAD (Ryan et al., 2014; PDQ Adult Treatment Editorial Board, 2018). At the time of diagnosis, PAAD usually has reached an advanced stage and has often metastasized to distant regions of the body. In recent years, most of the research on pancreatic cancer has focused on exploring specific tumor markers for PAAD. There are several evidences showing that the expression of long non-coding RNAs (lncRNAs) in PAAD is abnormal (Shi et al., 2018; Liu et al., 2020; Shuai et al., 2020). LncRNAs might, therefore, potentially become new markers for diagnosis and treatment of PAAD and could also be an important biological indicator for the prognosis of PAAD.

Long non-coding RNAs are a kind of RNAs with more than 200 nt in length; these RNAs cannot encode proteins. Most of the lncRNAs are transcripts of RNA polymerase II and are stored in nucleus and cytoplasm. Recent studies have confirmed that lncRNAs have unique structure and biological function and participate widely in gene regulation (Shuai et al., 2020). They play regulatory roles in the process of chromosome X silencing, genomic imprinting, chromatin modification, transcriptional activation, transcriptional interference, and intranuclear transport. LncRNAs regulate gene transcription through both cis- and trans- modes. In the cis-regulation mode, an lncRNA combines with unchained chromatin grooves or with unchained DNA strands or affects the splicing of an adjacent RNA. In the trans-regulation mode, an lncRNA binds to promoters and enhancers of distal RNAs, or acts as scaffold and recruits protein complexes, or regulates binding proteins or other RNA activities in the cytoplasm or nucleus in a dose-dependent manner.

Cancer susceptibility candidate 8 (CASC8 or CARLo-1) is located on 8q24 chromosome, in which MYC and several other lncRNAs are also located. MYC is known to be a key contributor to the development of several human tumors. Recent studies have revealed that the lncRNAs originating from the human 8q24 locus play important roles in MYC regulation. CARLo-5 was found to act in the cis-regulation mode to mediate chromatin looping between the MYC promoter and its enhancers. It forms multiple long-range chromatin interactions and mediates interaction with the MYC promoter and a well-characterized 8q24 enhancer, MYC335. It might be possible that CASC8 has similar functions because it is also involved in the development of several human tumors. Moreover, CASC8 polymorphisms (rs1447295 and rs10505477) have been reported as risk factors for some cancer types, such as gastric, hepatic, and breast cancers (Cui P. et al., 2018; Wu et al., 2019; Zhang et al., 2020). However, not much is known about the alteration of CASC8 expression in PAAD. Based on the information available in four cancer databases (starBase, cBioPortal, circlncRNAnet, and UCSC Xena), we analyzed the CASC8 expression and its mutations, and the effect of this lncRNA on the survival in PAAD. We also analyzed the competing endogenous RNA (ceRNA) network, and CASC8 polymorphisms to unravel the mechanism underlying its role in the development of PAAD (Wu et al., 2018; Zhang et al., 2020). We finally evaluated the expression level of CASC8 in PAAD tissues and paired adjacent normal pancreatic tissues collected from 13 Chinese patients after surgical resection.

## Materials and Methods

### Analysis of Cancer Susceptibility Candidate 8 Expression and Competing Endogenous RNA Network Using the starBase Database

For starBase^1^, the data for expression of genes in cancers were downloaded from The Cancer Genome Atlas (TCGA) project via Genomic Data Commons Data Portal. This database has data from PAAD-related studies for 178 samples. Differential expression analysis and survival analysis for CASC8 were performed. The expression of CASC8 and other genes are presented by FPKM (fragments per kilobase of transcript sequence per millions base pairs sequenced). FPKM is currently the most commonly used method for estimating gene expression levels.

The starBase database was also used to speculate the ceRNA pair of CASC8 and the co-target mRNA and miRNAs. Using 178 PAAD samples from TCGA, we analyzed the correlation between the expression of CASC8, H19, SMAD7, and miR-671, which would help in the understanding their expression pattern in PAAD and in the speculation of their interaction mechanism.

### Analysis of Cancer Susceptibility Candidate 8 Mutations Using the cBioPortal Database

The cBioPortal database^2^ was used to collect the data for PAAD cases. Data for five studies on PAAD and two studies on pancreatic neuroendocrine tumors (pNETs) were collected. In this database, mutations of CASC8 were analyzed based on 1142 PAAD samples.

### Analysis of Cancer Susceptibility Candidate 8 Enrichment Using the circlncRNAnet Database

The circlncRNAnet database^3^ is an integrated web-based resource for mapping functional networks of long or circular forms of non-coding RNAs. It was used to analyze the enrichment of CASC8 in PAAD. The PAAD data were downloaded from TCGA project.

### Analysis of Cancer Susceptibility Candidate 8 Polymorphisms Using the UCSC Xena Database

The UCSC Xena database^4^ is a genome-related database established by bioinformatics researchers at the University of California, Santa Cruz. It contains about 200 public datasets including TCGA, International Cancer Genome Consortium (ICGC), TARGET, Genotype-Tissue Expression (GTEx), and Cancer Cell Line Encyclopedia (CCLE). The association between CASC8 polymorphism and risk of pancreatic cancer was analyzed using 223 PAAD samples from TCGA.

### Collection of Patient and Tissue Samples

Fresh PAAD tissues and paired adjacent non-tumor tissues were collected from surgically resected specimens obtained from 13 Chinese patients at the Peking Union Medical College Hospital (PUMCH), Beijing, China. The tissue samples were histopathologically confirmed by three independent pathologists. Informed consent was obtained from the patients before surgery. The study was approved by the Ethics Committee of PUMCH.

### RNA Extraction and RT-qPCR

Total RNA was extracted using the TRIzol reagent (Invitrogen, China) according to the manufacturer’s instructions. The complementary DNA (cDNA) was synthesized using the SuperScript III Reverse Transcriptase Kit (Invitrogen, China). Real-time PCR was performed with Power SYBR Green PCR Master Mix (TransGen Biotech, China) on the ABI 7500 fast real-time PCR system. The amplification reaction included the following steps: 95°C for 10 min, followed by 40 cycles of 95°C for 15 s and 60°C for 1 min. GAPDH was used as an internal control for mRNA, and the relative expression level of mRNAs was calculated by the relative quantification (2^–ΔΔCT^) method. The primer sequences are listed in Table 1.

**TABLE 1:** Primers used for qRT-PCR.

	Forward primer (5′–3′)	Reverse primer (5′–3′)
CASC8	GCAGTGAGCCAAGGAGCAAT	AACCGCAACACTGGTTGTGT
GAPDH	GGCAGTGATGGCATGGACTGT	CCTTCATTGACCTCAACTAA

## Results

### Cancer Susceptibility Candidate 8 Is Upregulated in Pancreatic Adenocarcinoma

**FIGURE 1:**
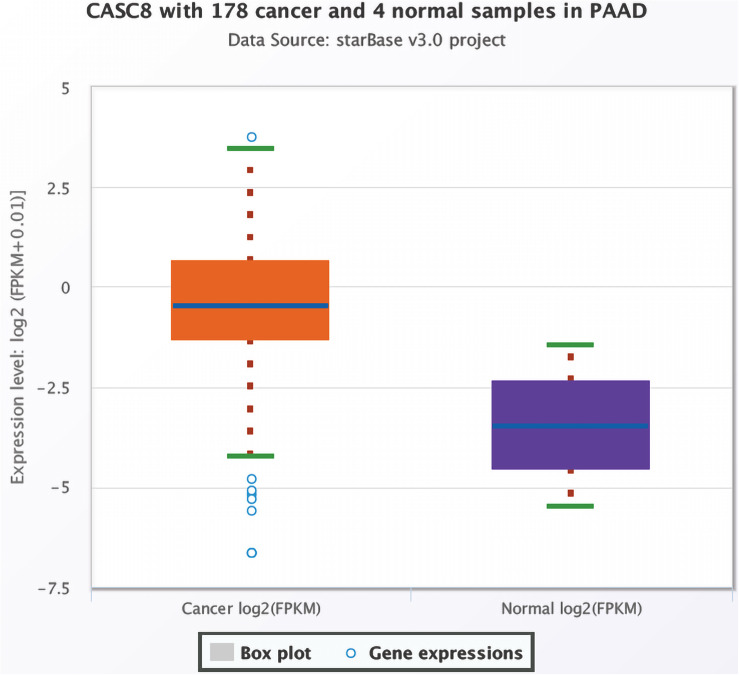
Differential expression analysis of cancer susceptibility candidate 8 (CASC8) in pancreatic adenocarcinoma (PAAD) and normal pancreatic tissues. Each point represents a sample, and the value is log2 FPKM of CASC8 expression.

Based on data for 178 cancer and four normal samples present in TCGA, the expression of CASC8 was found to be significantly upregulated in PAAD [FPKM, fold change = 8.71, *P* = 0.0014, false discovery rate (FDR) = 0.04] (Figure 1). Changes in the expression levels ranked third among 32 cancer types, just lower than in cholangiocarcinoma and lung squamous cell carcinoma.

### High Expression of Cancer Susceptibility Candidate 8 Is Associated With Shorter Overall Survival Time

**FIGURE 2:**
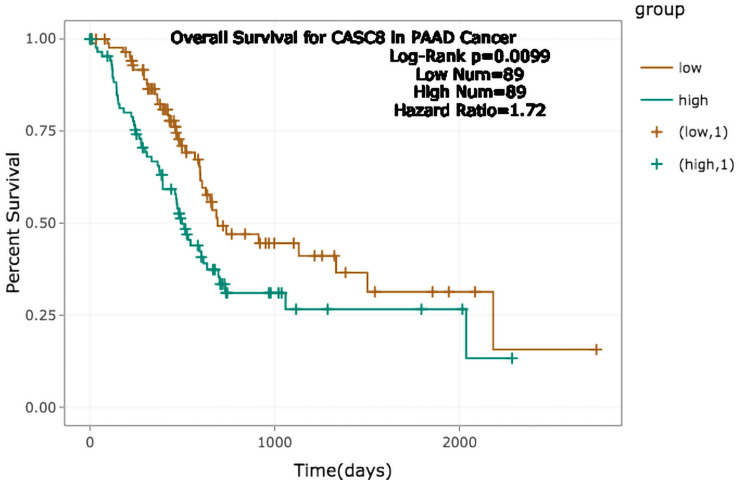
Relationship between cancer susceptibility candidate 8 (CASC8) expression and survival time of pancreatic adenocarcinoma (PAAD) patients.

A total 178 samples were divided into low and high CASC8 expression groups, according to the expression median. The survival analysis showed that the prognosis of low CASC8 expression group was better than that of its high expression counterpart [*P* = 0.0099, hazard ratio (HR) = 1.72] (Figure 2).

### Cancer Susceptibility Candidate 8 Is Amplified in 6% of Pancreatic Adenocarcinoma Patients and Is Related With a Shorter Survival Time

**FIGURE 3:**
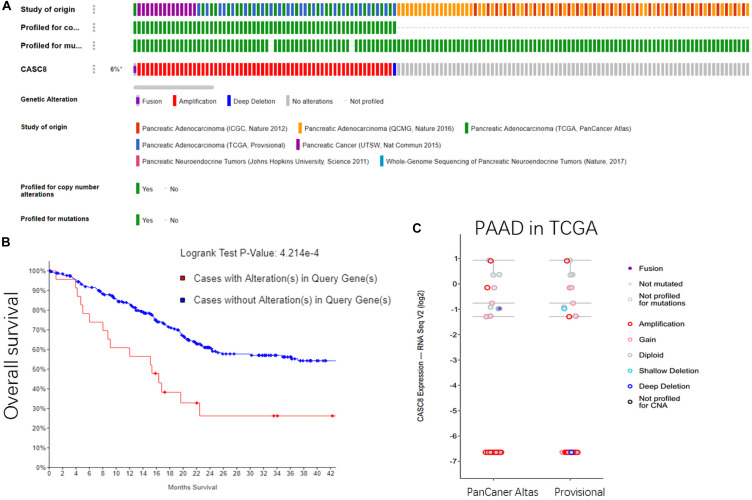
Gene alterations in cancer susceptibility candidate 8 (CASC8). **(A)** Summary for CASC8 studies. **(B)** Relationship between alterations in CASC8 and survival time. **(C)** CASC8 expression level for different types of alterations.

In all the 1142 samples in cBioPortal database, the copy number variants were detected in 62 of the cases (6%). Sixty cases were of CASC8 amplification, one case was of deep deletion, and another one was fusion expression of CASC8 with FAM49B. These gene alterations were reported in three studies, including a pancreatic cancer study in UCSC (14 amplification cases); a PAAD study in TCGA, PanCancer Atlas (24 cases); and PAAD study in TCGA, Provisional (24 cases) (Figure 3A). However, no alterations in CASC8 expression were found in the two datasets of pNETs.

The overall survival analysis showed that the cases with CASC8 alterations had a shorter survival time than those without such alterations (Figure 3B). Most of the samples with CASC8 amplification had the same expression level as was observed for the samples with no mutations, except for four cases (Figure 3C). Based on these evidences, the amplification of CASC8 might not function in enhancing the expression of CASC8 but might play a role in other ways that are involved in the progression of PAAD. Enrichment analysis of CASC8 differential expression samples may provide clues for these related pathways.

### Cancer Susceptibility Candidate 8 Enrichment Analysis

**FIGURE 4:**
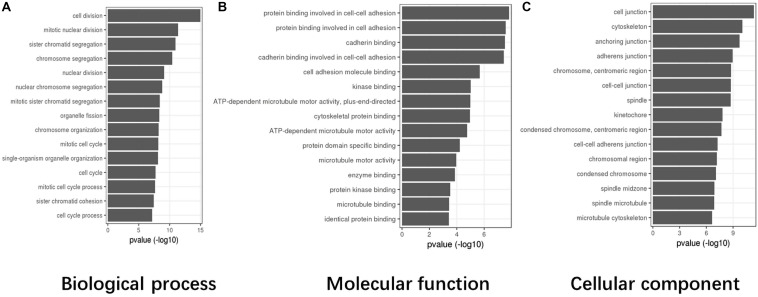
Gene Ontology (GO) enrichment analysis for cancer susceptibility candidate 8 (CASC8) (top 15). **(A)** Top 15 enrichment terms in biological process (BP) categories. **(B)** Top 15 enrichment terms in molecular function (MF) categories. **(C)** Top 15 enrichment terms in cellular component (CC) categories.

Based on TCGA data, the differentially expressed genes in the abnormal CASC8 samples were significantly enriched in “cell division,” “mitotic nuclear division,” “sister chromatid segregation,” “chromosome segregation,” and “nuclear division” in the biological process categories; “protein binding involved in cell–cell adhesion,” “protein binding involved in cell adhesion,” “cadherin binding,” “cadherin binding involved in cell–cell adhesion,” and “cell adhesion molecule binding” in the molecular function categories; and “cell junction,” “cytoskeleton,” “anchoring junction,” “adherens junction,” and “chromosome, centromeric region” in the cellular components categories (Figure 4).

**FIGURE 5:**
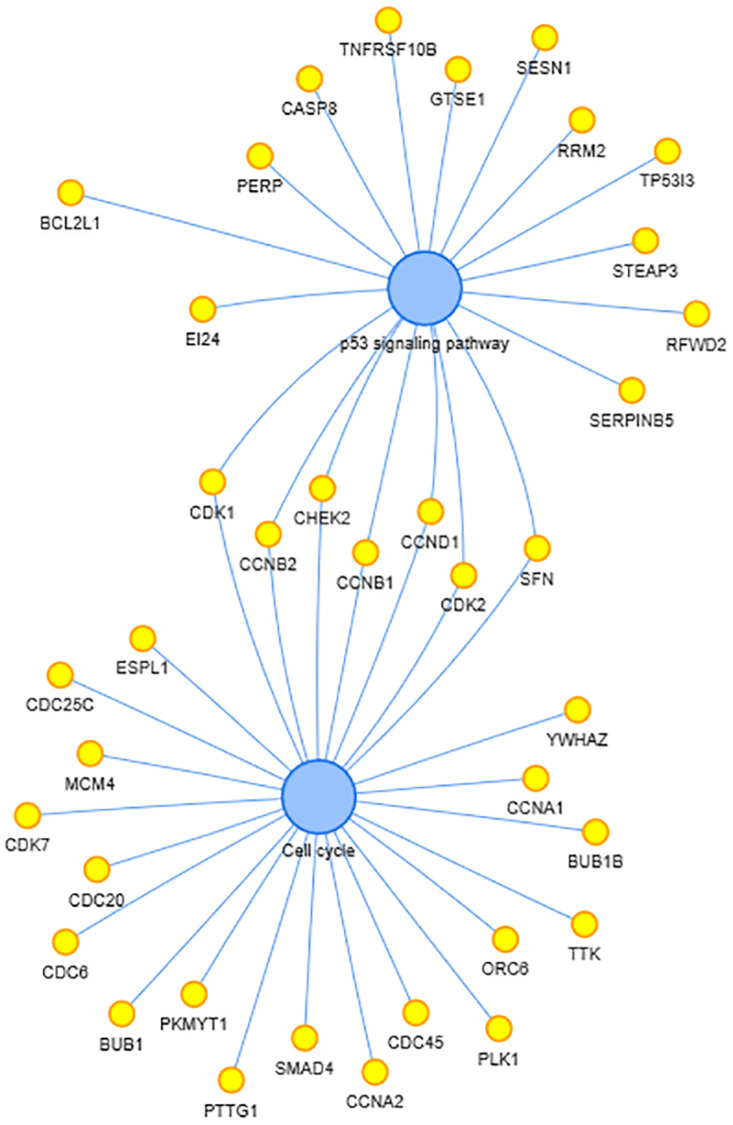
Kyoto Encyclopedia of Genes and Genomes (KEGG)-enriched pathways and related genes. The two blue dots in the center represent the enriched pathway and the yellow dots beside represent the enriched genes.

In the Kyoto Encyclopedia of Genes and Genomes (KEGG) enrichment analysis, two pathways, including p53 signaling pathway and cell cycle, were enriched. The co-expressed genes in these two pathways and interactions are shown in Figure 5. P53 are thought to be critical in pancreatic cancer development and progression, as well as SMAD7. CASC8 may be involved in the cell cycle, proliferation, and apoptosis of PAAD cells.

### H19 Interacts With Cancer Susceptibility Candidate 8 and miR-671

**FIGURE 6:**
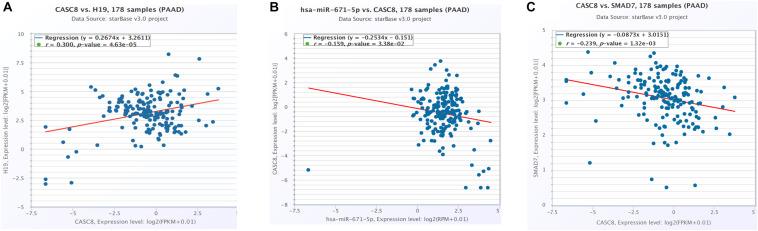
Cancer susceptibility candidate 8 (CASC8) correlation analysis with competing endogenous RNA (ceRNA)-H19 **(A)**, miR-671 **(B)**, and SMAD7 **(C)**. Each point represents a sample, and the value of *X*/*Y*-axis is log2 FPKM of corresponding gene expression.

**FIGURE 7:**
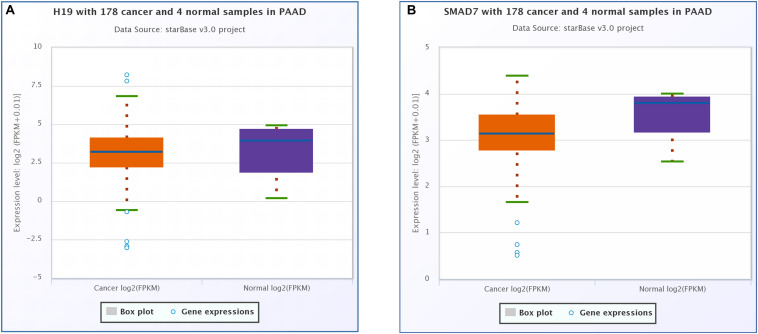
Differential expression analysis of **(A)** H19 and **(B)** SMAD7 in pancreatic adenocarcinoma (PAAD) and normal pancreatic tissues. Each point represents a sample, and the value is log2 FPKM of corresponding gene expression.

CeRNA network analysis identified H19 as a pair of CASC8 in PAAD using starBase (178 samples). H19 is an lnRNA that is differentially expressed in several tumors and participates in tumorigenesis. In cancer progression, H19 has a dual role as an oncogene or a tumor suppressor gene, depending on the cancer type and developmental stage. The correlation analysis showed that the CASC8 expression was changed more than that of H19 in 178 samples of PAAD, and they correlated positively with each other (Figure 6A). Unexpectedly, there was little difference between cancer patients and healthy people with regard to the H19 expression (Figure 7A).

Six miRNAs were predicted to interact with CASC8 and H19, including hsa-miR-599, hsa-miR-4761-5p, hsa-miR-3064-5p, hsa-miR-6504-5p, hsa-miR-4761-3p, and hsa-miR-671-5p. Hsa-miR-671-5p scored the highest among these lncRNAs, and further analysis showed that the expression of CASC8 was significantly negatively correlated with that of miR-671 (Figure 6B).

### Correlation Analysis Between Cancer Susceptibility Candidate 8 and SMAD7

SMAD7 expression had been shown to be implicated in the progression of fibrosis in different organs such as pancreas (Kuang et al., 2006; Li et al., 2016). A relationship between the CASC8, SMAD7 genes, and risk of colorectal cancer were analyzed in a meta-analysis including 90 studies (168,471 cases and 163,223 controls) (Yao et al., 2015). So we analyzed the correlation between CASC8 and SMAD7 in PAAD. In TCGA, the expression of SMAD7 was significantly downregulated in PAAD (Figure 7B). Correlation analysis showed that CASC8 was significantly negatively correlated with SMAD7 (Figure 6C). These results suggested the interaction between SMAD7 and CASC8 may be similar to that of miR-671.

### High Copy Number Segment of Cancer Susceptibility Candidate 8 Is Associated With Low Survival Probability

**FIGURE 8:**
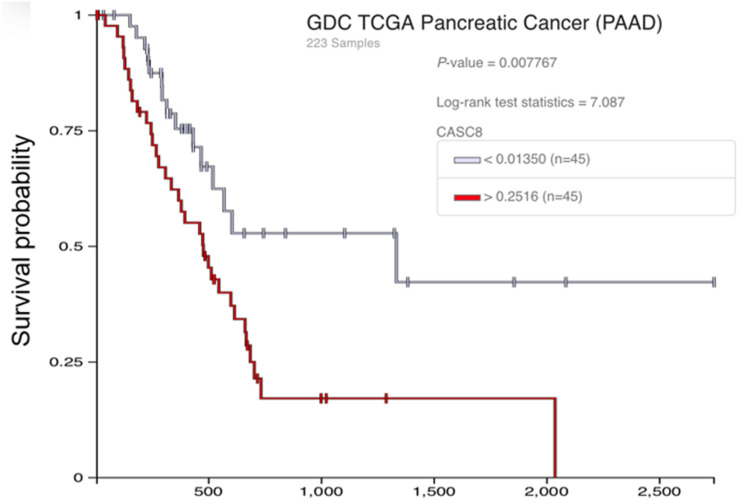
Relationship between the copy number segment (CNS) of cancer susceptibility candidate 8 (CASC8) and survival time of pancreatic adenocarcinoma (PAAD) patients.

We further analyzed the effect of CASC8 gene polymorphism on pancreatic cancer. Although there is a lack of information on single-nucleotide polymorphisms (SNPs) in these databases, we analyzed the copy number segment (CNS) instead. Forty-five samples were categorized in the high CNS group (CNS > 0.2516) and 45 samples in the low CNS group (CNS < 0.0135). Statistical analysis showed that there were significant differences in the probability of survival between the two groups (Figure 8). After 1000 days, the probability of survival in the low CNS group was two times more than in the high CNS group.

Gene expression analysis was also performed between CASC8 high expression group (*n* = 43, FPKM > 15.18) and low expression group (*n* = 46, FPKM < 13.15) by Xena database (Supplementary Figure S1). The survival probability of CASC8 high expression group was significantly different with low expression group (*P* < 0.001). The result is consistent with the CASC8 CNS analysis.

### qPCR Validation of Cancer Susceptibility Candidate 8 Expression in Chinese Population

Among the 13 PAAD tissue samples from Chinese patients, there were five tumor samples with significantly high CASC8 expression, in which four samples were highly differentiated and the cancer stages (according to *AJCC Cancer Staging Manual*/Edition 8th) were 2B(T2), 1B(T2), 1B(T2), and 2A(T3) separately as the expression level of CASC8 decreased gradually. The other sample was poorly differentiated, and its cancer staging was 1A(T1c). The expression of CASC8 in other six tumor samples was low, and four samples among these were moderately differentiated, one sample was highly differentiated, and its cancer stage was 1A(T1b). One sample was poorly differentiated and was in stage 3(T2).

**FIGURE 9:**
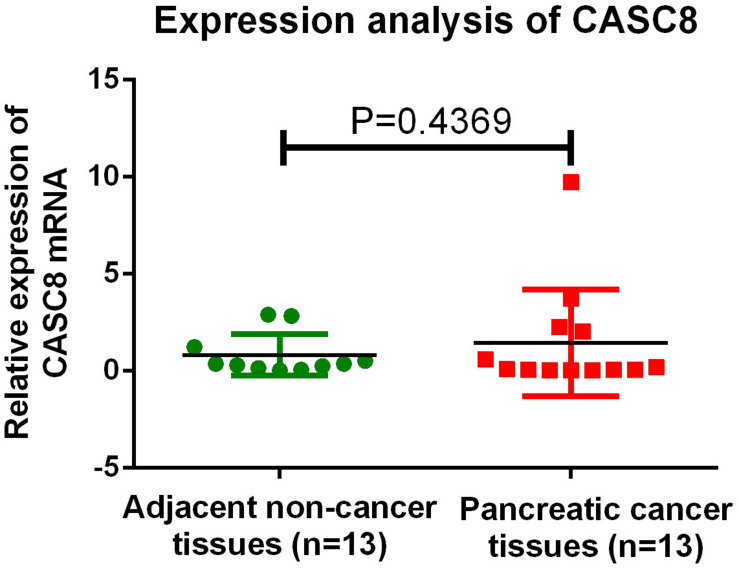
qPCR validation of cancer susceptibility candidate 8 (CASC8) expression in 13 Chinese pancreatic adenocarcinoma (PAAD) patients. Tukey’s multiple comparison test was used to analyze the differences among 13 samples from two groups and to estimate the level of significance.

The expression of CASC8 was not detected in the two normal tissue samples, possibly owing to operating problems or some issue with the sample itself; unfortunately, not enough tissue was left for retesting. Overall, in all the samples, the expression level of CASC8 in cancer tissues was 1.76-fold relative to that in normal tissues (1.46 vs. 0.83, *P* = 0.4369, Figure 9).

In summary, in the selected PAAD samples, 80% (4/5) of the highly differentiated and 50% (1/2) of the poorly differentiated cases showed high expression levels of CASC8, whereas all (4/4) the moderately differentiated samples showed low expression of CASC8.

## Discussion

In this study, we analyzed 178 PAAD samples in the starBase database and identified that higher expression of CASC8 in the PAAD tissues (fold change = 8.71, *P* = 0.0014, FDR = 0.04) was related with poor prognosis. Based on 1142 samples in the cBioPortal database, about 6% of the PAAD patients were identified to have alterations in CASC8. Most of the alterations of CASC8 in the PAAD samples were gene amplifications (Figure 3A), which have not yet been fully studied in cancer. However, in most of the CASC8 amplification cases, the expression of CASC8 was not increased compared with that in their counterparts. This indicated that the high expression of CASC8 was not caused by its amplification. Interestingly, no CASC8 alterations were found in the two datasets of pNETs, which indicated the changes and effects of CASC8 might be specific for PAAD. In PAAD, there might be some pathways, such as the MYC enhancer, to enhance the expression of CASC8 at the transcriptional level. Moreover, the gene amplification was found to correlate with the survival time of PAAD patients. It might be possible that the amplification of CASC8 gene results in the formation of a special 3D structure that prevents MYC from enhancing the expression from its promoter. Further studies are needed to provide evidence for this hypothesis. However, our study confirmed CASC8 as a key factor in PAAD, and CASC8 amplification may be involved in the development of PAAD.

In recent years, the clinical significance of CASC8 has been extensively studied, especially in cancer. Most of the studies on CASC8 have focused on its polymorphism. CASC8 rs10505477 polymorphism was found to be greatly associated with gastrointestinal toxicity in small cell lung cancer (SCLC) and could serve as a possible risk marker for diagnosing lung cancer and could be used to forecast the response and toxicity of platinum-based treatment in lung cancer patients (Hu et al., 2016; Zhang et al., 2020). The rs10505477 is located in the intron of CASC8 which overlaps with the POU5F1B (POU class 5 homeobox 1 pseudogene 1) gene. A study on a Chinese population found that rs10505477 GA heterozygote in CASC8 was associated with a significantly lower survival rate in gastric cancer (Zhang et al., 2020). A meta-analysis for CASC8 in cancer showed that, compared with the individuals carrying the rs10505477 TT genotype, those with the TC or CC genotype had a decreased risk of cancer, whereas for rs7837328, the GA and AA genotypes were associated with increased risks of cancer as compared with the GG genotype. These two polymorphisms might be associated with the risk of cancer (Hu et al., 2016; Zhang et al., 2020). A meta-analysis including 22 studies (47,003 cases and 45,754 controls) evaluated the rs6983267 of CASC8 in colorectal cancer, suggesting that the rs6983267 G > T polymorphism is a risk factor in Asians, Europeans, and Americans with European ancestry (Haerian et al., 2017). The rs10505477 may disrupt the correlation between CASC8 and the promoter of POU5F1BB, which was found to be a putative cancer susceptibility gene (Kastler et al., 2010; Ma et al., 2015). Although there is a lack of information on SNP in these databases, we analyzed the CNS of CASC8 instead. The results showed that high CNS of CASC8 was associated with low probability of survival. All these results indicate that CASC8 may play an important role in the progression of PAAD.

We also identified the ceRNA network between CASC8 and H19 in the present study. The overexpression of H19 has been documented in breast, bladder, colon, and other types of cancers (Cui Z. et al., 2018; Wang et al., 2020). Aberrant expression of H19 in these tumors is related to increased cell proliferation and invasion and reduced apoptosis as a result of direct inactivation of the tumor suppressor gene, p53, and increase ID2 expression (Zhao et al., 2017; Zheng et al., 2019). H19 also functions as a ceRNA. It antagonizes the functions of miR-141 and miR-22 and leads to de-repression of their shared target gene, β-catenin, which eventually activates the Wnt/β-catenin pathway (Liang et al., 2016). In this study, we found that CASC8 was significantly positively correlated with H19 and predicted that CASC8 and H19 competitively bind to the microRNA-miR-671. In breast cancer, miR-671-5p is a tumor-suppressor miRNA in breast tumorigenesis and works by repressing Forkhead Box M1 (FOXM1), which is an oncogenic transcription factor, and causes epithelial-to-mesenchymal transition (EMT) in MDA-MB-231 breast cancer cells (Tan et al., 2016). In Gene Ontology (GO) enrichment analysis, the cell binding and adhesin terms were enriched (Figure 4). During EMT, cell binding and adhesion are weakened. Therefore, with higher expression, CASC8 may work as a sponge to bind miR-671, de-repress FOXM1, and promote EMT in PAAD. The relationship with H19 and miR-671 indicates that CASC8 may be a potential therapy target in PAAD.

Hu et al. (2017) showed that CASC8 is significantly downregulated in bladder cancers and is associated with the advanced stage of bladder cancer patients. They identified FGFR1 as the target of CASC8 and found that it is involved in the conversion of pyruvate into lactate. However, we observed an opposite trend in the CASC8 expression levels in PAAD. Yao et al. (2015) identified the relationship between the CASC8, SMAD7, and the risk of colorectal cancer. Although there is no such findings in PAAD at present, and we found that CASC8 was significantly negatively correlated with SMAD7. Our findings suggest that there are different mechanisms underlying the CASC8 function between these cancers, and further functional experiments should be performed.

The limitations of this study are as follows: first, the relationship between tumor staging and CASC8 is not included for data limitation; second, the population in the databases is mainly from western countries, which may cause systematic deviations. Fortunately, our validation test in Chinese patients was consistent with the analytical findings and may decrease such risk. Third, the sample size in our study is small. We strictly screened our patients and samples, and tissue samples were identified by three pathologists, which took a lot of time. In addition, there were two normal tissue samples wherein we failed to detect CASC8 expression, possibly owing to operating problems or the problem of the sample itself. Additionally, the individual differences and heterogeneity of PAAD might also affect the results.

In summary, we comprehensively analyzed four popular databases and found the alterations and changes in the expression pattern of CASC8 in PAAD, as well as the CNS of CASC8, correlate with PAAD prognosis. Furthermore, through ceRNA network analysis, we predicted that CASC8 may work as a sponge to bind miR-671, competing with H19, and activate the EMT process in PAAD. Finally, we validated the high expression levels of CASC8 in Chinese PAAD tissues, which strongly supported our analytical findings. Despite the limitations, our findings indicate that CASC8 may be a novel prognostic marker and a potential target for therapy of PAAD. More rigorous studies are warranted to confirm this hypothesis.

## Data Availability Statement

Publicly available datasets were analyzed in this study. These data can be found here: starbase (http://starbase.sysu.edu.cn), Cbioportal database (http://www.cbioportal.org/), circlnc RNAnet database (http://app.cgu.edu.tw/circlnc/), UCSC Xena database (http://xena.ucsc.edu/).

## Ethics Statement

The studies involving human participants were reviewed and approved by the Research Ethics Committee of Peking Union Medical College Hospital. The patients/participants provided their written informed consent to participate in this study.

## Author Contributions

YiW and YX contributed to the preparation of the research, literature review, and writing of the manuscript. YaW and XL collected the data. YY and WW provided ideas and recommendations and reviewed the manuscript. All the co-authors saw the manuscript and agreed to its contents.

## Conflict of Interest

The reviewer (HZ) declared a shared affiliation, with no collaboration, with several of the authors to the handling Editor at the time of review. The remaining authors declare that the research was conducted in the absence of any commercial or financial relationships that could be construed as a potential conflict of interest.

## Footnotes

1 http://starbase.sysu.edu.cn

2 http://www.cbioportal.org/

3 http://app.cgu.edu.tw/circlnc/

4 http://xena.ucsc.edu/

## Supplementary Material

FIGURE S1 Differential expression of CASC8 and probability of survival in the UCSC Xena database.
