# Supplementary figures and images for: High Cancer Susceptibility Candidate 8 Expression Is Associated With Poor Prognosis of Pancreatic Adenocarcinoma: Validated Analysis Based on Four Cancer Databases
Source: Front Cell Dev Biol. 2020 Jun 4;8:392. doi: 10.3389/fcell.2020.00392 (PMC7287184; doi:10.3389/fcell.2020.00392)

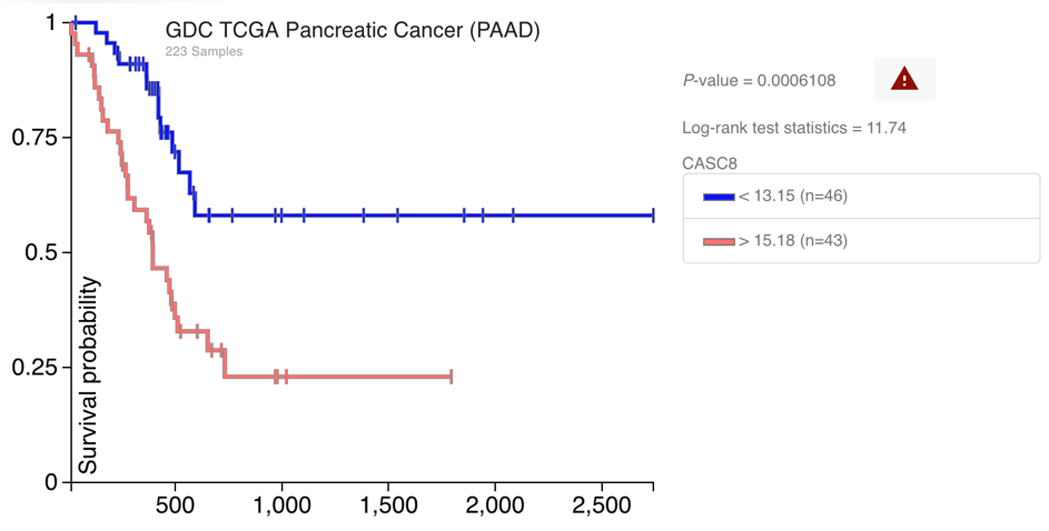

Supplement: FIGURE S1 — Differential expression of CASC8 and probability of survival in the UCSC Xena database. [file Image_1.TIF]
